# Self-care of Caregivers: Self-Compassion in a Population of Dutch Medical Students and Residents

**DOI:** 10.15694/mep.2020.000222.1

**Published:** 2020-10-08

**Authors:** Juliet Godthelp, Maaike Muntinga, Theo Niessen, Piet Leguit, Tineke Abma

**Affiliations:** 1 Amsterdam UMC; 2 Canisius Wilhelmina Hospital; 3 Platform-ECG Foundation

**Keywords:** self-compassion, medical education, burnout, student well-being, gender.

## Abstract

This article was migrated. The article was marked as recommended.

**Objectives:** The incidence of burnout in medical students and residents continues to outpace that of the general population. Self-compassion, a concept in the study of well-being, may moderate against adverse mental health outcomes. The aim of this study is to extend prior research by investigating self-compassion levels in relation to sociodemographic variables and self-reported burnout in Dutch medical students and residents.

**Methods:** We used a cross-sectional survey design. After inclusion, 295 participants completed the online survey. Self-compassion was measured using the Self-Compassion Scale Short-Form. Self-defined burnout symptoms were measured using a single-item measure. Data were analysed using multiple linear regression.

**Results:** Being male was associated with having higher levels of self-compassion (β=0.131, p<.001) as well as being of higher age (β=0.175, p<.001). Reporting burnout was negatively associated with self-compassion (β=-.412, p<.001).

**Discussion:** This study substantiated previous research linking low self-compassion to burnout, and showed a potential increased vulnerability of young and female students. Further investigation of causality and the processes underlying self-compassion development are needed to investigate whether self-compassion interventions can enhance the well-being of medical students and residents.

## Introduction

Clinical wards are increasingly understood as risk environments for future doctors’ mental and emotional health. Studies suggest that medical students and residents experience higher rates of burnout than their peers ( Conijn, Boersma and van Rhenen, 2015; Fares *et al.*, 2016; Van Esch and Soomers, 2018) and point to various potential negative outcomes of student distress, including life dissatisfaction, sickness absence, poor performance, discontinuation of medical studies, depression and suicide ( Dyrbye, Thomas and Shanafelt, 2005). At a professional level, burnout-related symptoms, such as emotional exhaustion, depersonalization and reduced personal accomplishment, are reported as implicit barriers for compassionate and effective patient care ( Dyrbye *et al.*, 2010; Neumann *et al.*, 2011).

The high burnout rates among future doctors are often related to contextual factors inherent to their professional environment ( Dev, Fernando and Consedine, 2020). Dutch medical training is characterized by a high work intensity due to heavy patient loads and long, irregular working days ( Van Esch and Soomers, 2018). Other than a challenged work-life integration, lack of autonomy and emotional burden are found to contribute to higher levels of stress ( Prins *et al.*, 2007; Conijn, Boersma and van Rhenen, 2015). But despite similar environmental stressors, some medical students tend to be more vulnerable to burnout than others ( Dunn, Iglewicz and Moutier, 2008). In general, such differences in burnout risk are translated to a variety of coping strategies and personality factors, such as rumination, self-efficacy and optimism ( Alarcon, Eschleman and Bowling, 2009).

In clinical medical education, self-compassion may be an insightful construct in explaining differences in vulnerability in particular. Encouraged to become excellent, empathic and self-reflective professionals, medical students are inclined to perfectionism and the associated intolerance for ambiguity, mistakes and loss ( Nedrow, Steckler and Hardman, 2013). Self-compassion rather than self-criticism may lead to improved self-care and personal understanding in this demanding and competitive environment, putting personal experiences into perspective ( Neff, 2003). Institutional emphasis on empathic care without providing a compassionate learning environment may not only result in increased emotional exhaustion but also hinder mutually satisfying patient-physician interactions (Fernando *et al.*, 2016; Tierney, Ozer and Perry, 2018). This catch-22 has been broadly recognized.

So far, a myriad of compassion interventions has been implemented and evaluated ( Kirby, 2017) which are empirically supported by a wealth of research linking self-compassion to psychological well-being, life-satisfaction and social connectedness ( MacBeth and Gumley, 2012; Marsh, Chan and MacBeth, 2018). Few studies, however, investigated these associations between self-compassion, well-being and performance in the setting of medical education. Dev, Fernando and Consedine (2020) recently found that greater self-compassion predicted lower burnout and better quality of life in a sample of 383 medical students, with female students reporting lower levels of self-compassion. In a smaller sample of paediatric residents, self-compassion was positively associated with resilience ( Olson, Kemper and Mahan, 2015). Self-compassion was also found to be significant in explaining mastery approach and avoidance goals in medical students by Babenko and Oswald (2018).

To replicate and extend this primarily preliminary research on self-compassion in medical training, this study investigated self-compassion levels of Dutch medical students and residents in relation to sociodemographic characteristics and self-defined burnout. By doing so, we aimed to contribute to a better understanding of the self-compassion construct and it’s applicability within Dutch medical education. Based on previous literature indicating gender and burnout differences with regard to self-compassion levels, we hypothesized that female gender and burnout would predict lower levels of self-compassion. Because age and ethnicity differences in self-compassion have not been systematically studied among young adults but have been identified as potential substantive factors ( Yarnell *et al.*, 2015), we did not advance the exploration of those factors into specific hypotheses.

## Methods

### Participants

*Setting:* The study population consisted of bachelor students, master students and residents of all eight Dutch medical schools which are traditionally connected to academic medical centers, teaching hospitals attached to a university. Similar to most other European medical schools, their curricula consist of a three year program of preclinical studies (bachelor), followed by three years of clinical rotation (master) ( Patrício and Harden, 2010). To enter the bachelor program, candidates have to complete a competitive qualitative selection procedure. The ratio of female to male students entering is approximately 2 to 1, in line with the feminization of medicine worldwide ( Bleakley, 2013). To pursue specialization, post-graduates can either apply for a job as a pre-specialty doctor or research scientist (assistant not in training, ANIOS), or start a residency at the desired specialty training site as assistant in training (AIOS).

**Figure 1.**
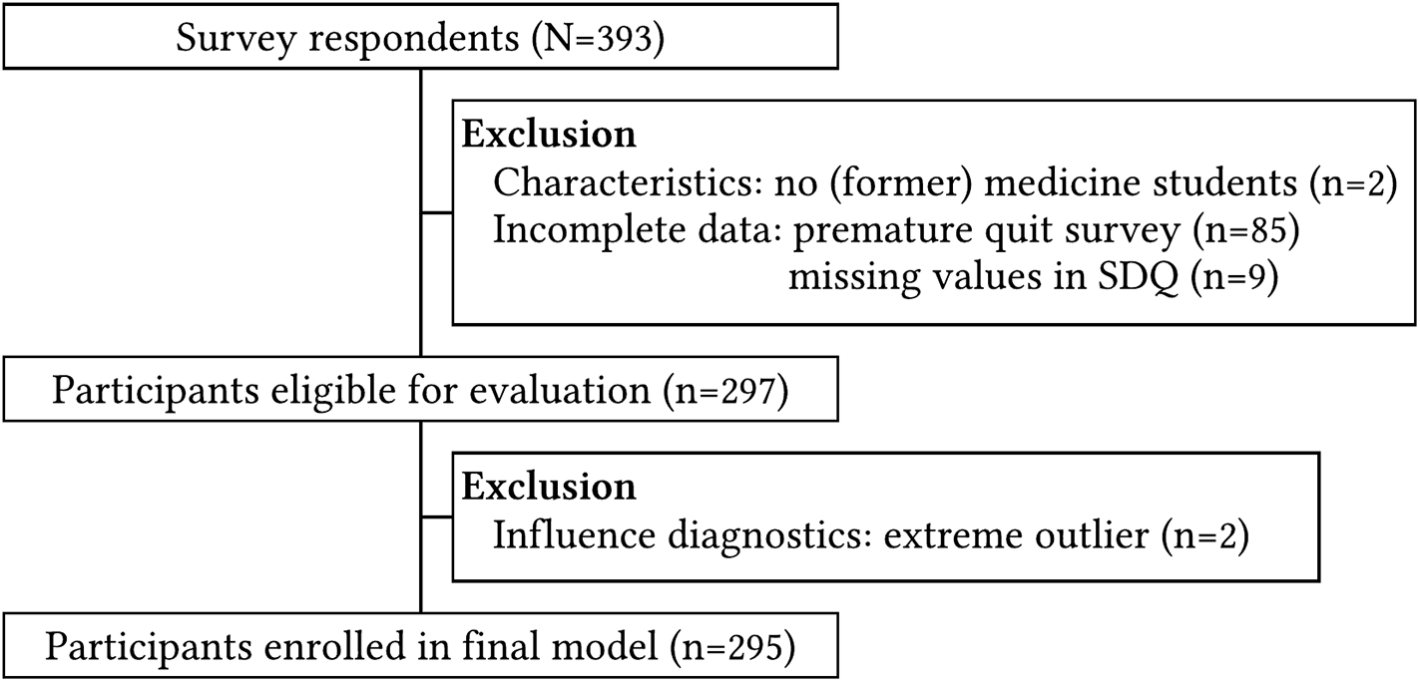
Flowchart of inclusion (N, number of respondents; n, number of selected participants; SDQ, sociodemographic questionnaire).

*Recruitment:* Participants were recruited through the networks of medical student associations and through senior clinical staff involved in this study. They were invited to fill out an online survey by means of a link, to which a total of 393 responded. Two respondents were excluded because they were not part of the study population. 94 respondents (24%) were excluded because they did not complete the survey or showed missing values in the sociodemographic questionnaire (See Figure 1). The final number of participants was 295. The majority of study participants were female (81%) and identified as having a native Dutch background. Other ethnicity backgrounds included European, Hindustani, Moroccan, American, Peruvian, Japanese and Turkish. Three AIOS currently working in Dutch hospitals graduated at universities outside of the Netherlands. The mean age was 26 years.

### Procedure

Data was collected from July 10 to August 22, 2018. We used a composite questionnaire, consisting of a self-compassion measure, a self-defined burnout measure and sociodemographic items.

### Measures

Participants completed a demographic survey, including gender (male, female, other), age, identification with ethnical background (native Dutch, other), medical study progress (Bachelor student **,** Master student **,** ANIOS, AIOS) and faculty of medical studies.

Self-compassion was measured with the Dutch version of the Self-Compassion Scale Short-Form (SCS-SF). The SCS-SF correlates highly with the 26-item Self-Compassion Scale (SCS-LF) when examining the total scores (r≥0.97) ( Raes *et al.*, 2011). We used the short form to ensure lower respondent burden and increase response rate without affecting the quality of the investigation. The SCS-SF consists of twelve items measured along a 7-point Likert scale (range almost never - almost always) with mean score of 48.1 in the normative population of Dutch university students ( Raes *et al.*, 2011). Items include statements such as “When something painful happens I try to take a balanced view of the situation”, or “When I’m feeling down I tend to obsess and fixate on everything that’s wrong”. Six of the 12 items are reverse-scored.

To further minimize the survey material, we used a single-item measure of self-defined burnout which was validated against the Maslach Burnout Inventory in over 1000 physicians (r=0.64-0.68, p<0.0001) ( Rohland, Kruse and Rohrer, 2004; Hansen and Girgis, 2010). Responses were scored on a five-point ordinal scale ranging from (1) *“*I enjoy my work. I have no symptoms of burnout” to (5) “I feel completely burned out and often wonder if I can go on. I am at the point where I may need some changes or may need to seek some sort of help *”.*This measure can be dichotomized as ≤ 2 (no symptoms of burnout) and ≥3 (1 or more symptoms of burnout) ( Dolan *et al.*, 2015).

### Data Analysis

Prior to descriptive statistics, self-compassion total scores were computed. Subsequently, analyses proceeded in two phases. First, all potential associations were assessed using Pearson’s correlations to investigate covariance (r≤.3 low; r=.3-.7 moderate correlation) and identify potential multicollinearity (r≥.8). Second, multiple linear regression was performed to quantify and test the effects of the sociodemographic variables and burnout on self-compassion. Even though Likert scale items are ordinal in nature, usage of parametric tests was justified because summative ratings of Likert scales can be treated as interval-level variables ( Carifio and Perla, 2008).

Multiple linear regression was conducted using a standard non-sequential entry approach. Age, gender ethnicity and burnout were used as independent variables to set up a parsimonious explanatory model of self-compassion. The variable study progress was not included because of suspected collinearity with age. After excluding a severe outlier (standardized residual >3.29), the results of tests on normality, linearity, homoscedasticity and multicollinearity indicated that the assumptions needed for multiple linear regression were met ( Field, 2013). Interaction terms were added and tested to detect potential inter-variable effects. The F-test, (adjusted) R-squared and t-test were used to study the goodness of fit of the final regression models. All statistics were tested two-tailed at an alpha level of .05 using SPSS version 23 (IBM Corporation, New York, NY, USA).

## Results/Analysis

Table 1 shows the general demographics, mean total score of the SCS-SF and the single-item burnout measure outcomes. The mean total SCS-SF score was 49.0 (range 19-78). Over half of the respondents indicated feeling occasionally stressed, while approximately a quarter of the respondents indicated experiencing symptoms of burnout.

**Table 1.** Characteristics of study population (n=295).

	Total
*Gender (N (%))* Female	240 (81.4)
*Age (mean (range) SD)*	26.0 (18-40) SD 4.5
*Ethnicity (N (%))* Native Dutch background Other	275 (93.2) 20 (6.8)
*Study Progress (N (%))* Bachelor student Master student ANIOS AIOS	72 (24.4) 80 (27.1) 54 (18.3) 89 (30.5)
*Faculty of Studies (N (%))* Erasmus Universiteit Rotterdam Maastricht University Radboud Universiteit Nijmegen Rijksuniversiteit Groningen Universiteit Leiden Universiteit Utrecht Universiteit van Amsterdam Vrije Universiteit Other	19 (6.4) 38 (12.9) 11 (3.7) 43 (14.6) 17 (5.8) 64 (21.7) 55 (18.6) 45 (15.3) 3 (1.0)
*Total Score SCS-SF (mean (range) SD)*	49.0 (19-78) SD 11.5
*Self-defined Burnout (N (%))* No symptoms of burnout Occasionally stressed One or more symptoms of burnout Symptoms won’t go away Totally burned out	52 (17.6) 166 (56.3) 52 (17.6) 12 (4.1) 13 (4.4)

Abbreviations: N, number; %, valid percentage; SD, standard deviation; ANIOS, post-graduate working as pre-specialty doctor or research scientist; AIOS, resident of specialty training sites; SCS-SF; Self-compassion Scale Short-Form.

Correlation analyses showed a low positive correlation between self-compassion and older age (r=.281, p<.001) and male gender (r=.242, p<.001). A moderate negative correlation was found between self-compassion and reporting symptoms of burnout (range 3-5) (r=-.344), p<.001). No significant correlation was found between self-compassion and ethnicity background. The AIOS level of study progress was weakly correlated to higher levels of self-compassion (r=.254, p<.001) but strongly correlated to older age (r=.782, p< .001) and being male (r=.261, p<.001). These results indicated collinearity between age and study progress conforming to our expectations.

The first multiple linear regression equation, comprising the variables self-defined burnout, age and gender, was found to be significant (F(7,287)=19.5, p<.001), explaining 32 percent of the variance of self-compassion (R ^2^=.323, adjusted R ^2^=.306) (see Table 2). Except for ethnicity background, all variables were significant predictors of self-compassion. On average, men scored 4.0 points higher on the SCS-SF. For each year of age, self-compassion increased .43 point on the SCS-SF. Participants who reported symptoms of burnout showed lower levels of self-compassion which decreased with 8.0 points on average per subsequent category of burnout except for category 5 (“totally burned out”). Addition of two-way (burnout*gender, burnout*age, gender*age, gender*ethnicity) and three-way (gender*ethnicity*age) interaction terms did not explain additional variance of self-compassion (p-values ranging from .45 to .87).

**Table 2.** Results from multiple linear regression analyses on the SCS-SF total score (n=295).

	Model 1	
	β	t
*Gender* Male	.134	2.64 *
*Age*	.169	3.29 **
*Self-defined Burnout* A B C D E	- -.389 -.422 -.411 -.303	--5.85 ** -6.64 ** -7.76 ** -5.61 **
*Ethnicity* Other	.061	1.246
R ^2^	.323	
Adjusted R ^2^	.306	
F	19.5 **	

SCS-SF, Self-Compassion Scale Short-Form; β, standardized B; t, t-test value; R ^2^, R squared; F, F-test value; Self-defined burnout; no symptoms of burnout (A), occasionally stressed (B), one or more symptoms of burnout (C), symptoms won’t go away (D), totally burned out (E).

* p < .01

** p < .001

## Discussion

This study quantified self-compassion in a sample of Dutch medical students and residents. The average total score on the SCS-SF corresponded with the average score of the normative population ( Raes *et al.*, 2011). Consistent with previous literature, reporting lower self-compassion levels was strongly associated with self-defined burnout, putting forward self-compassion as a potential explanatory factor in burnout risk in medical education. Theories framing this association suggest that self-compassion functions as a stress moderator by taking difficulties and personal shortcomings into perspective with an attitude of self-kindness, rather than over-identification and self-criticism ( Neff and Dahm, 2015). Self-compassion was also found to be a mediator in the relationship between mindfulness and well-being, including evidence suggesting that self-compassion is a stronger predictor of well-being than mindfulness alone ( Neff and Dahm, 2015; Evans *et al.*, 2018).

These findings may be relevant to medical education in particular. Implicit and explicit criteria related to medical professionalism lead to shared understandings around who is seen as a good physician, and who is not ( Van den Brink and Benschop, 2012). Self-care may implicitly be misconceived as being weak, passive and unprofessional, undermining self-improvement motivation ( Neff, 2003). Whilst research suggest that this perceived stigma and a fear of negative personal consequences may contribute to the limited number of burnout medical students seeking help ( Dyrbye *et al.*, 2015), fostering self-compassion may relate to more effective care-seeking and caregiving ( Hermanto and Charles Zuroff, 2016; Kirby, 2017). Although interventional studies are needed to determine causality, this suggests that cultivation of compassion for oneself and others could benefit students’ self-care practices as well as their learning outcomes.

Confirming our hypothesis, we found that female medical students reported significantly lower levels of self-compassion. An interaction between ethnicity and gender, comparable to the findings of a meta-analysis by Yarnell *et al.* (2015) reporting larger differences between men and women in more ethnically diverse samples, was not retrieved in this study. Gender-based differences may be explained by tendency in women to have a more ruminative coping style which relates to greater barriers towards self-compassion and burnout ( Neff, 2003; Neff and McGehee, 2009; Raes, 2010) but may also lay in values dominant in medical professionalism. Traditionally masculine qualities such as rationalism, oppositionalism, certainty and distance are highly regarded on the medical work floor, whereas more feminine qualities such as reconciliation and collaboration are considered less essential ( Bleakley, 2013). In the process of “fitting in”, female physicians may be more likely to ignore their own feelings and vulnerability ( Verdonk *et al.*, 2014). Even though the exact underlying mechanisms remain unclear, gender-based differences point out that interventions targeting self-compassion to enhance psychological well-being may be particularly effective in women. However, the majority of the variance of self-compassion is shared by gender and should not be overemphasized ( Yarnell *et al.*, 2015).

Consistent with previous research, younger respondents reported lower self-compassion ( Neff and McGehee, 2009). One explanation for this association is that undergraduate medical students are in a life phase of identity formation in which they are more likely to continually evaluate and compare themselves to others ( Ragelienė, 2016). Whilst age did not alter the significance of the association between low self-compassion and burnout, older residents still reported burnout symptoms. This suggests that self-compassion may be a prerequisite for resilience, yet its positive effects are mediated by increased demands from residents’ everyday work context. Further research is needed to explore this hypothesis.

### Limitations

This study furthered the field of medical student well-being research by quantifying students’ self-compassion and burnout levels during clinical medical education. We did, however, identify several limitations. First, we did not have enough power to reliably investigate the relationship between residential specialties, ethnicity and other aspects of diversity and self-compassion. This limits our ability to distinguish differences in self-compassion between majority and minority subgroups which may be at higher risk of burnout due to intentional and unintentional homogenizing influences in medical training ( Beagan, 2000).

In addition, the cross-sectional design did not allow for investigation of causality, limiting our ability to gain deeper insight in the relationship between self-compassion and burnout-related symptoms. Furthermore, our findings might have been prone to over- or underestimation as a result of response bias, such as social desirability or self-presentational biases ( Podsakoff, MacKenzie and Podsakoff, 2012) and selection bias due to the use of self-report measures. Varying participation may affect the generalizability of our findings, yet they are consistent with findings of longitudinal cohort studies and represent all medical faculties of the Netherlands.

## Conclusion

Despite its limitations, this study suggests self-compassion to be a valuable construct to gain insight in medical student well-being. The detected determinants of interpersonal differences, age and gender, could provide a handle for curricular integration of the concept, but further research is needed to investigate physiological and psychological processes that underlie self-compassion development. Longitudinal studies on self-compassion in medical education are needed to study causality between low self-compassion and burnout, and prove the ability of self-compassion interventions to improve the well-being of medical students and residents. Such studies could also tell more about the predictive value of self-compassion in relation to other student-level outcomes including compassion and learning outcomes. Additional clinometric evaluation of the SCS-SF questionnaire could provide insight in its usefulness as a screening or case finding tool.

Moreover, we need to study the effects of present work ethics in medical education and practice on student well-being. A sole focus on resilience training would suggest that an individual’s failure to cope results from personal incapacity rather than from dysfunctional educational system. Stigma related to self-care could severely hinder efforts to provide students with individual coping capital. In-depth qualitative research is essential to gain insight in student experiences and the processes that underlie the relationship between self-compassion, burnout and contextual factors. Extra attention should be paid to potentially vulnerable subgroups, such as female students and students of non-majority backgrounds, in order to add to our understanding of ways in which these groups can be empowered and supported ( Verdonk *et al.*, 2019).

## Take Home Messages

• Self-compassion is associated with reporting fewer symptoms of burnout in medical students and residents.
• Female and young medical students are especially at risk for lower levels of self-compassion.
• Qualitative and longitudinal studies on self-compassion training in medical education are needed to investigate processes underlying self-compassion development and prove the ability of compassion interventions to protect medical students and residents from burnout.

## Notes On Contributors

Juliet J. Godthelp, BSc (Cum Laude), is medical student and researcher, dept. Medical Humanities, Amsterdam UMC, location VUmc.

Maaike Muntinga **,** MSc, PhD is researcher and lecturer in Diversity in Health and Health Professions Education, dept. Medical Humanities, Amsterdam UMC, location VUmc.

Piet Leguit **,** MD, PhD, holds chair of ECG, former surgeon and former President Association Surgeons of the Netherlands.

Theo Niessen, MSc, PhD, Dean of Nursing Research at Canisius Wilhelmina Hospital, Nijmegen, the Netherlands.

Tineke A. Abma **,** MSc, PhD is deputy head of the dept. Medical Humanities, professor Participation & Diversity, research leader in Amsterdam Public Health research institute, Amsterdam University Medical Centres. Director of Leyden Academy on Vitality and Ageing, Leiden.
