# Supplementary material for: Self-care of Caregivers: Self-Compassion in a Population of Dutch Medical Students and Residents
Source: MedEdPublish (2016). 2020 Oct 8;9:222. [Version 1] doi: 10.15694/mep.2020.000222.1 (PMC10699363; doi:10.15694/mep.2020.000222.1)
Supplement: Peer review history

# Reviewer report: Reviewer response for version 1

**DOI:** 10.21956/mep.20152.r31132

**Published:** 2021-01-09

**Authors:** Ken Masters

**Affiliations:** 1 Sultan Qaboos University

This review has been migrated. The reviewer awarded 5 stars out of 5 An interesting paper dealing with self-compassion among Dutch medical students and residents. This is a useful topic, as burnout among medical students is well-documented, and the concept of self-compassion is posited as a counter to this phenomenon.The background is clearly explained, and the study has been well-conducted, using validated tools. The results are fairly presented, and the limitations acknowledged.All-round, a solid and useful piece of research, not only for researchers in the field, but also for all medical educators.I do have one query on the interpretation of the correlation. A (r=.281, p

Reviewer Expertise:

NA

No decision status is available

# Reviewer report: Reviewer response for version 1

**DOI:** 10.21956/mep.20152.r31131

**Published:** 2020-10-13

**Authors:** P Ravi Shankar

**Affiliations:** 1 American International Medical University

This review has been migrated. The reviewer awarded 4 stars out of 5 This is an interesting study on self-care and self-compassion among caregivers. The authors introduce the concept of self-compassion which is an important one for medical students and doctors. The description is well structured and detailed information about the study methodology is provided. Self-care and resilience are important characteristics and have become even more important during the ongoing pandemic. The article will be of interest to a broad group of medical educators.

Reviewer Expertise:

NA

No decision status is available
